# Analysis of the Relevance of the Ultrasonographic Features of Papillary Thyroid Carcinoma and Cervical Lymph Node Metastasis on Conventional and Contrast-Enhanced Ultrasonography

**DOI:** 10.3389/fonc.2021.794399

**Published:** 2021-12-23

**Authors:** Tian Xue, Chang Liu, Jing-Jing Liu, Yan-Hong Hao, Yan-Ping Shi, Xiu-Xiu Zhang, Yan-Jing Zhang, Yu-Fang Zhao, Li-Ping Liu

**Affiliations:** Department of Ultrasound, First Hospital of Shanxi Medical University, Taiyuan, China

**Keywords:** papillary thyroid carcinoma (PTC), cervical lymph node metastasis, conventional ultrasound, contrast-enhanced ultrasound (CEUS), nomogram

## Abstract

**Background:**

Preoperative prediction of lymph node metastases has a major impact on prognosis and recurrence for patients with papillary thyroid carcinoma (PTC). Thyroid ultrasonography is the preferred inspection to guide the appropriate diagnostic procedure.

**Purpose:**

To investigate the relationship between PTC and cervical lymph node metastasis (CLNM, including central and lateral LNM) using both conventional ultrasound (US) and contrast-enhanced ultrasound (CEUS).

**Material and Methods:**

Our study retrospectively analyzed 379 patients diagnosed with PTC confirmed by surgical pathology at our hospital who underwent US and CEUS examinations from October 2016 to March 2021. Individuals were divided into two groups: the lymph node metastasis group and the nonmetastasis group. The relationship between US and CEUS characteristics of PTC and CLNM was analyzed. Univariate and multivariable logistic regression methods were used to identify the high-risk factors and established a nomogram to predict CLNM in PTC. Furthermore, we explore the frequency of CLNM at each nodal level in PTC patients.

**Results:**

Univariate analysis indicated that there were significant differences in gender, age, tumor size, microcalcification, contact with the adjacent capsule, multifocality, capsule integrity and enhancement patterns in CEUS between the lymph node metastasis group and the nonmetastasis group (all *P*<0.05). Multivariate regression analysis showed that tumor size ≥1 cm, age ≤45 years, multifocality, and contact range of the adjacent capsule >50% were independent risk factors for CLNM in PTC, which determined the nomogram. The diagnostic model had an area under the curve (AUC) of 0.756 (95% confidence interval, 0.707-0.805). And calibration plot analysis shown that clinical utility of the nomogram. In 162 PTC patients, the metastatic rates of cervical lymph nodes at levels I-VI were 1.9%, 15.4%, 35.2%, 34.6%, 15.4%, 82.1%, and the difference was statistically significant (*P*<0.001).

**Conclusion:**

Our study indicated that the characteristics of PTC on ultrasonography and CEUS can be used to predict CLNM as a useful tool. Preoperative analysis of ultrasonographical features has important value for predicting CLNM in PTCs. The risk of CLNM is greater when tumor size ≥1 cm, age ≤45 years, multifocality, contact range of the adjacent capsule >50% are present.

## Introduction

Thyroid carcinoma is one of the most common malignancies of the endocrine system (1). The incidence of thyroid carcinoma has been increasing in recent years (2). It can be divided into four types: medullary carcinoma, undifferentiated carcinoma, follicular carcinoma and papillary carcinoma. Of all the thyroid carcinomas diagnosed, papillary thyroid carcinoma (PTC) is the most common subtype, accounting for 80%-90%. PTC develops indolently, but 30% to 80% of patients with PTC demonstrate highly aggressive diseases, such as cervical lymph node metastasis (3). The presence of cervical lymph node metastasis (CLNM) is well associated with an increased risk of recurrence and affects the postoperative survival rate in select patient populations (4, 5). The early diagnosis of lymph node metastasis can guide clinicians to perform aggressive therapeutic neck dissection. It can also improve the surgical management of patients with clinical lymph node-negative papillary thyroid carcinoma to avoid additional prophylactic cervical lymph node dissection and reduce the risks of surgery (6). Therefore, the preoperative diagnosis of CLNM plays a critical role in the surgical strategy and postoperative outcomes of patients with PTC (7).

Ultrasonography is a convenient and simple imaging technique used to study the characteristics of PTC, and many small lesions can be detected with the increase in the resolution of ultrasound (US) diagnostic equipment and the emergence of new US technology. The metastasis of PTC to cervical lymph nodes can be evaluated in a timely manner with US. However, conventional ultrasound has certain limitations in the diagnosis of thyroid cancer metastasis. Previous studies (8) have demonstrated the feasibility of contrast-enhanced (CEUS) in the diagnosis of thyroid malignant nodules. CEUS is widely used to study tumor microcirculation, which is an important feature used to differentiate between benign and malignant tumors and determine the potential to metastasize (9). To our knowledge, few reports (10) have mentioned the value of CEUS in PTC patients for predicting CLNM. In this study, to improve the preoperative diagnosis of CLNM and assist the clinical selection of more meaningful individual lymph node dissections, we used both US and CEUS to investigate the relationship between imaging characteristics and metastasis to cervical lymph nodes, and to establish a clinical diagnostic model to help surgeons predict CLNM. In contrast to previous studies, tumor contact with the capsule was regarded as an important factor to indicate the invasion and metastasis of tumor cells. We not only observed the continuity of the capsule with CEUS but also quantified and grouped the capsule invasion.

## Material and Methods

### Patients

The present study’s design and protocol were approved by the Ethics Committee of our hospital, and all patients signed informed consent before CEUS examination and surgery. From October 2016 through March 2021, we used a surgical pathology database to identify 379 patients with PTC in our study. All patients underwent conventional US and CEUS imaging before surgery. There were 69 males and 310 females aged 18 to 70 years. Of those, 162 patients had CLNM, and 217 patients had no metastasis. The inclusion criteria for the patients were as follows: (a) US and CEUS performed <two weeks before surgery, (b) pathologic types of nodules all confirmed as PTC, (c) age 18 years or older, and (d) no previous thyroid operation history. The exclusion criteria were as follows: (a) nodules that were not confirmed to have PTC by histopathological examination, (b) thyroid nodules that had previously undergone ablation or other treatment, (c) patients who were allergic to sulfur hexafluoride microbubbles (SonoVue) or had a coagulation disorder, and (d) pregnancy.

### Methods

Ultrasonographic examinations were performed with a GE LOGIQ E9 color Doppler ultrasound diagnostic instrument (GE Medical Systems, Milwaukee, WI, USA) equipped with an ML6-15 (6-15 MHz) linear array transducer for conventional US and a 9 L (2-9 MHz) linear array transducer for CEUS.

Conventional US examination: The patient was placed on a bed in the supine position with the neck region fully exposed, and then conventional ultrasound images of thyroid nodules were acquired by scanning the thyroid and lymph nodes in various regions of the neck with a probe to determine the target nodules. Characteristics recorded during the US examination included nodule location, size, echogenicity, boundary, margin, shape (taller than wide or wider than tall), microcalcification, whether the nodule was in contact with the adjacent thyroid capsule and its extent of contact, and multifocality.

CEUS examination: Once the target nodule was selected during the examination, the best section was chosen to display both the nodule and peripheral gland at the same time. The patients were instructed to breathe quietly without talking, swallowing, coughing, etc. The contrast agent used in this study was Sulphur hexafluoride microbubble (SonoVue^®^, Bracco, Milan, Italy). The patients were given 1.5 ml of contrast agent as a bolus through a 20-gauge cannula placed in the antecubital vein, followed by a 5-ml saline flush. Then, imaging was recorded with CEUS continuously for 90 s. Real-time images were stored for later analysis. The enhancement patterns of the nodule and the continuity of the thyroid capsule were compared with the surrounding thyroid parenchyma.

Imaging analysis: Based on postoperative pathology, the patients were divided into two groups: the lymph node metastasis group and the nonmetastasis group. General clinical features, conventional US features of nodules, and CEUS features were included.

1. The general clinical characteristics included gender (male, female) and age (≤ 45 years, >45 years).

2. The conventional US features included tumor size, echogenicity, boundary, margin, shape, microcalcification, extent of contact with the adjacent capsule, and multifocality.

Based on the extent of contact with the adjacent capsule, the patients were divided into two groups: a contact group and a noncontact group. A multisection scan was performed, and contact was defined as no normal thyroid tissue found between the border of the cancer nodule and the thyroid capsule; otherwise, it was defined as noncontact. For the extent of contact with the adjacent capsule, based on the contact proportion of the nodule with its perimeter, the patients were divided into four subgroups: no contact, contact extent <25%, contact extent between 25% and 50%, and contact extent >50%.

3. CEUS enhanced intensity was classified as hypoenhancement, isoenhancement, or hyperenhancement in the arterial phase with respect to the surrounding normal thyroid parenchyma. Capsule integrity was studied with CEUS, and based on this, the patients were divided into two subgroups: a continuous membrane group and an interrupted membrane group. The membrane was defined as continuous if the thyroid capsule showed a line-like intact structure using contrast enhancement; otherwise, it was defined as interrupted.

### Statistical Analysis

SPSS 22.0 statistical software (SPSS Inc, Chicago, IL, USA) was used for statistical analysis. The count data were analyzed by the *χ^2^* test. Continuous quantitative data were analyzed by *t* test, and rank data were analyzed by the rank sum test. Variables that showed univariate significance with CLNM were entered into a multivariate logistic regression analysis to ascertain the independent risk factors. A value of *P <*0.05 was considered statistically significant. We evaluated the performance of the diagnosis model and determined the 95% confidence intervals of the AUC. The nomogram was constructed using R software version 3.5.1 (R foundation for statistical computing, Vienna, Austria. URL http://www.R-project.org) and conducted internal verification. Calibration curve and concordance index (C-index) were used to verify the accuracy and consensus degree of the prediction model. The area under the curve (AUC) was estimated from the ROC curve (receiver operating characteristic curve).

## Results

A total of 379 PTC patients were included in this study (69 males and 310 females). The patients were aged 18 to 70 years, with an average age of 45.12 ± 10.91 years. Of them, 162 patients were in the lymph node metastasis group, including 37 males and 125 females, with an average age of 42.04 ± 11.51 years. There were 217 patients in the nonmetastasis group, including 32 males and 185 females, with an average age of 47.42 ± 9.85 years.

We converted continuous variables (age and tumor size) into classification variables according to ROC curves. The best cutoff values of tumor size and age in PTC for discriminating with and without CLNM were 45 years and 1.15 cm, respectively. However, referring to the relevant literature (11, 12), thyroid carcinoma with a tumor size less than 1 cm was defined as papillary thyroid microcarcinoma, so we divided the tumor size into two groups with a cutoff of 1 cm.

### Valuable Indicators of US and CEUS Between the Lymph Node Metastasis Group and the Non-Metastasis Group

The basic characteristics of the patients and conventional ultrasound features of PTCs are shown in **Table 1**. There were significant differences in gender, age, tumor size, microcalcification, contact with the adjacent capsule, and multifocality between the PTC nodule metastasis group and the nonmetastasis group by the *χ^2^* test (*P <*0.05). There were no significant differences in echogenicity, boundary, margin or shape.

**Table 1:** Comparison of characteristics of PTCs between lymph node metastasis group and non-metastatic group.

Features		Metastasis group	Non-metastatic group	Metastasis rate (%)	*χ^2^*	*P* value
		n=162	n=217			
Gender	Male	37	32	53.6	4.080	0.043
	Female	125	185	40.3
Age	≤45	105	80	56.8	28.997	<0.001
	>45	57	137	29.4
Tumor size	<1	59	139	29.8	28.393	<0.001
	≥1	103	78	56.9
Echogenicity	Isoechoic/Hyperechoic	12	9	57.1	1.883	0.170
	Hypoechoic	150	208	41.9
Margin	Regular	21	41	33.9	2.385	0.123
	Irregular	141	176	44.5
boundary	Well-defined	22	38	36.7	1.076	0.300
	Ill-defined	140	179	43.9
shape	Taller than wide	60	93	39.2	1.305	0.253
	Wider than tall	102	124	45.1
Microcalcification	Yes	121	124	49.4	12.497	<0.001
	No	41	93	30.6
Contact with the adjacent capsule	Yes	126	143	46.8	6.354	0.012
	No	36	74	32.7
Multifocality	Yes	91	94	49.2	6.134	0.013
	No	71	123	36.6

The extent of tumor-capsule contact between the CLNM group and the nonmetastasis group was significantly different based on the rank sum test (*P*<0.001). The extent of tumor-capsule contact in the metastasis group was larger than that in the nonmetastasis group, and the larger the contact range of the adjacent capsule was, the higher the CLNM rate (**Table 2**).

**Table 2:** Comparison of the extent of tumor-capsule contact between the metastasis group and non-metastatic group.

Group		Metastasis group	Non-metastatic group	Metastasis (%)	*Z*	*P* value
		n=162	n=217			
The extent of tumor-capsule contact	None	36	74	32.7%	-4.578	<0.001
	<25%	41	76	35.0%		
	25%-50%	41	49	45.6%		
	>50%	44	18	77.0%		

**Figure 1:**
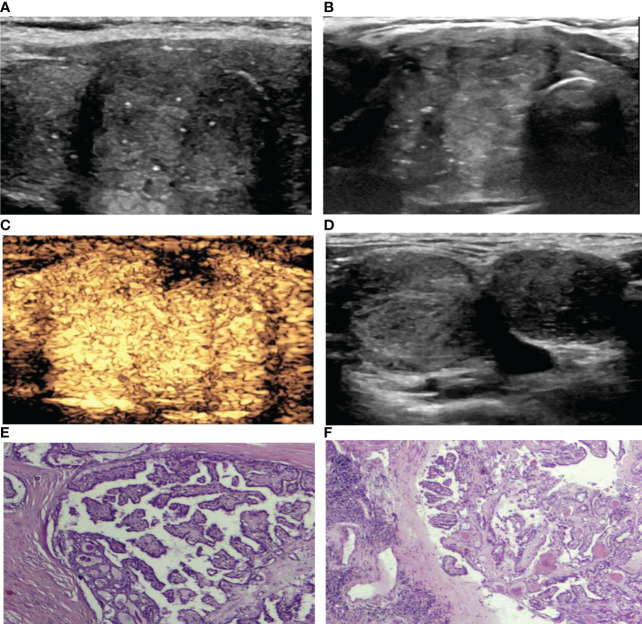
A 39-year-old woman with PTC. **(A)** Preoperative ultrasound images showing that the size of the tumor was approximately 3.85*3.27*2.01 cm in the right lobe, and the boundary was ill-defined, with microcalcifications. **(B)** Transverse sonogram showing that the contact range with the capsule was approximately >50%. **(C)** CEUS showing heterogeneous hyperenhancement in the nodule with interrupted thyroid capsule integrity (no enhancement area). **(D)** Lymph nodes were metastatic in levels III-IV of the right neck with the normal structures disappearing. **(E)** Surgical pathology confirming PTC (HE, hematoxylin-eosin, original magnification, ×40). **(F)** Surgical pathology confirming cervical lymph node metastasis from PTC (hematoxylin-eosin, original magnification, ×40).

**Figure 2:**
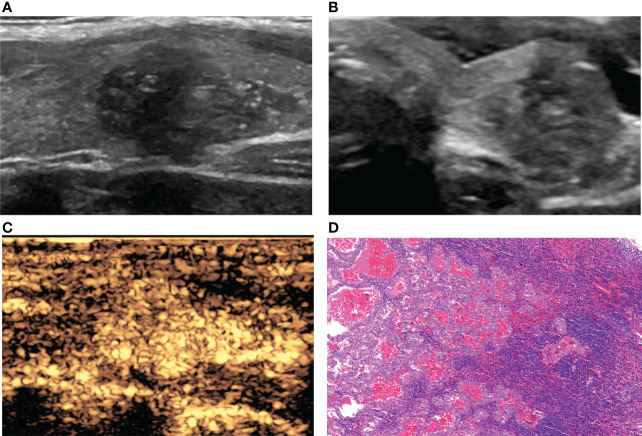
A 36-year-old woman with PTC. **(A)** Preoperative ultrasound images showing that the size of the tumor was approximately 1.81*1.30*1.09 cm in the left lobe, and microcalcification was detected. **(B)** Transverse sonogram showing that the contact range with the capsule was >50%. **(C)** CEUS showing hyperenhancement of the whole nodule. **(D)** Confirmation by surgical pathology as CLNM in ipsilateral level VI (HE, original magnification, ×100).

There was a significant difference in the integrity of the capsule on CEUS between the two groups (*P*<0.05). There were significant differences between the hyperenhancement group and the hypoenhancement group or the isoenhancement group (*P<*0.05), while there was no significant difference between the isoenhancement group and the hypoenhancement group (**Figures 1**, **2** and **Tables 3**, **4**).

**Table 3:** CEUS features of PTCs in lymph node metastatic group and non-metastatic group.

Characteristics		Metastatic group	Non-metastatic group	Metastasis (%)	χ2	*P* value
		n=162	n=217			
Enhancement	hypo	109	159	40.7	8.632	0.013
	iso	32	48	40.0		
	hyper	21	10	67.7		
Integrity of the capsule	continuous	95	171	35.7	18.015	<0.001
	Interrupt	67	46	59.3

**Table 4:** CEUS enhancement extent of PTCS in lymph node metastasis group and non-metastatic group.

Enhancement	Metastasis group	Non-metastatic group	*χ^2^*	*P* value
	n=162	n=217		
Hyper	21	10	8.286	0.004
Hypo	109	159		
Iso	32	48	0.012	0.914
Hypo	109	159		
Hyper	21	10	6.892	0.009
Iso	32	48		

**Figure 3:**
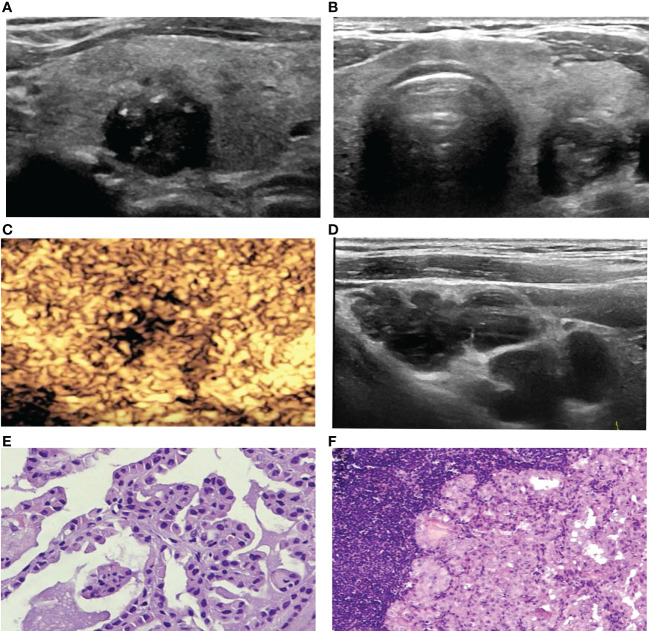
A 67-year-old woman with PTC. **(A)** Preoperative ultrasound images showing that the size of the tumor was approximately 1.2*1.0*1.3 cm in the left lobe, the margin was irregular, taller than wide, and calcification was detected. **(B)** Transverse sonogram showing that the contact range with the capsule was approximately 25%-50%. **(C)** CEUS showing heterogeneous hypoenhancement in the nodule. **(D)** Metastatic lymph node showing that the normal structure disappeared, presenting cystic changes. **(E)** Surgical pathology confirming PTC (HE, original magnification, ×100). **(F)** Surgical pathology confirming cervical lymph node metastasis from PTC, (HE, original magnification, ×100).

**Figure 4:**
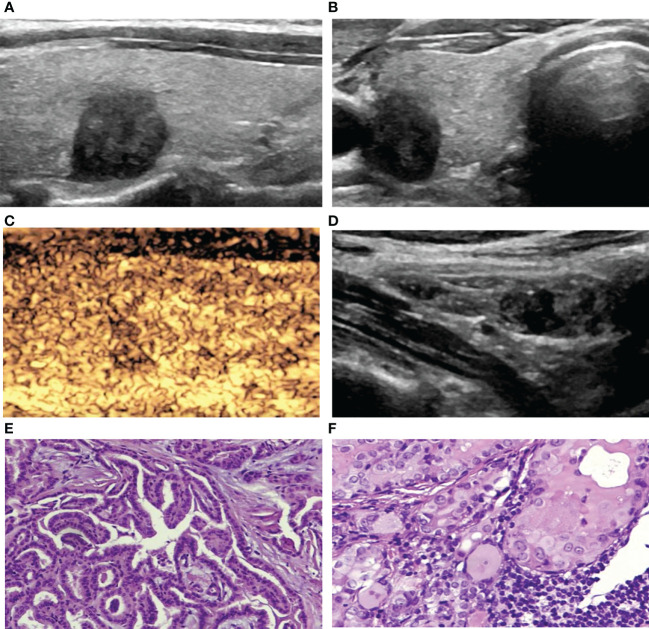
A 42-year-old woman with PTC. **(A)** Preoperative ultrasound images showing that the size of the tumor was approximately 1.00*0.77*1.03 cm in the right lobe with hypoechogenicity, an irregular margin, a poorly defined boundary and a taller-than-wide shape. **(B)** Transverse sonogram showing that the contact range with the capsule was approximately 25%-50%. **(C)** CEUS showing heterogeneous hypoenhancement in the nodule. **(D)** Metastatic lymph node showing that the normal structure disappeared, and microcalcification was detected inside. **(E)** Surgical specimen confirming PTC (HE, original magnification, ×100). **(F)** Surgical specimen confirming cervical lymph node metastasis from PTC (HE, original magnification, ×100).

The significantly different variables included patient gender, age, tumor size, microcalcification, contact with the adjacent capsule, multifocality, capsule integrity and enhancement patterns on CEUS. We included all the significant factors in univariate analysis into multivariate logistic regression, and finally screened out four independent predictors, multivariate logistic regression analysis showed that age ≤45 years (odds ratio, OR=3.846), tumor size ≥1 cm (OR=2.526), contact range of the adjacent capsule >50% (OR=3.023), and multifocality (OR=2.370) were independent risk factors for CLNM in PTC patients (**Figures 1**–**4** and **Table 5**).

**Table 5:** Logistic regression analysis of cervical lymph node metastasis in PTC.

Variable	*β*	*S.E*	Wald	*P*	*OR*	*OR* 95% CI	
						Lower limit	Upper limit
Age	1.347	0.241	31.272	<0.001	3.846	2.399	6.117
Tumor size	0.926	0.280	10.978	0.001	2.526	1.460	4.369
The contact range of the adjacent capsule >50%	1.106	0.422	6.876	0.009	3.023	1.322	6.911
Multifocality	0.863	0.241	12.826	<0.001	2.370	1.478	3.800
Constant	-2.133	0.320	44.500	<0.001	0.118		

β is the regression coefficient; S.E is the standard error; Wald is the chi-square value; OR is the odds ratio.

### Risk Factors-Based Nomogram Development

**Figure 5:**
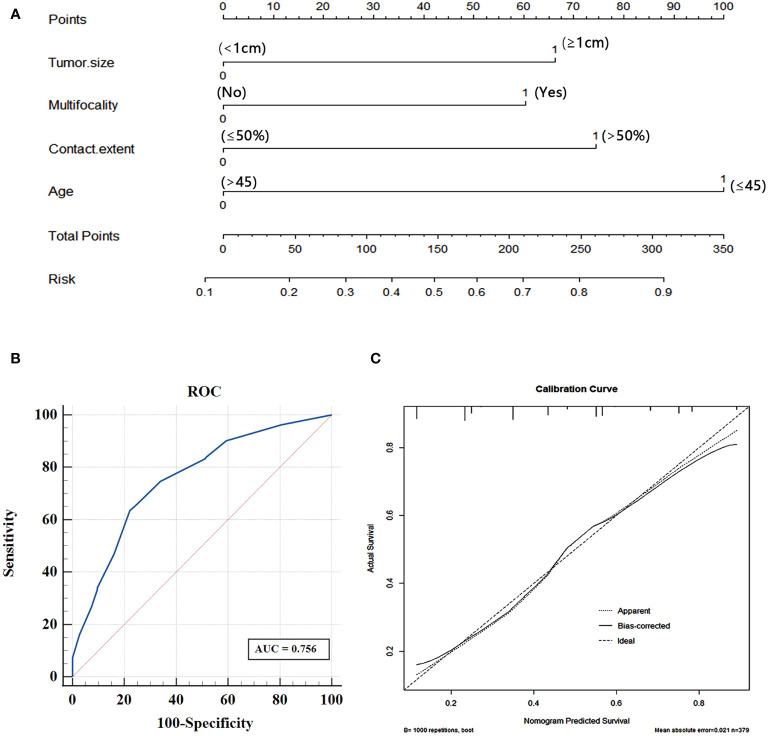
**(A)** Nomogram for predicting CLNM in PTC patients based on four risk factors. **(B)** The ROC curve and AUC of the nomogram; ROC, receiver operating characteristics. **(C)** Calibration plots of nomogram for predicting CLNM (internal validation set).

To visualize the model data, we developed a nomogram based on clinical risk characteristics to assess the degree of individual risk (**Figure 5A**). This nomogram integrated four risk factors (tumor size, age, multifocality, contact range of the adjacent capsule >50%), of which age was one predominant predictor of CLNM. Based on the scores for each variable, add them up to obtain the total score, locating it on the total point scale, and determine the individual’s probability of predicting CLNM. We used AUC to access the model performance. The AUC was 0.756(95% confidence interval, 0.707-0.805, **Figure 5B**). This nomogram had a good C-index of 0.756. The calibration plot also showed that the nomogram prediction was in good agreement with actual lymph node metastasis (**Figure 5C**), mean absolute error =0.021.

### Levels Status of Cervical Lymph Node Metastasis in PTCs

Among the 162 PTC patients with CLNM, there were significant differences among different CLNM levels (*P*<0.05). Using paired comparisons and the Bonferroni method to analyze the different CLNM levels, the highest metastasis rate was found at level VI, which was significantly different from the other levels (*P*<0.05). The metastasis rate at level I was the lowest, and there was a significant difference compared with the other levels (*P*<0.05). There was no significant difference between level II and level V (*P*>0.05), but there was a significant difference between level II and the other metastasis levels (*P*<0.05). There was no significant difference between level III and level IV (*P*>0.05), but there was a significant difference between level III and the other metastasis levels (*P*<0.05; **Table 6**).

**Table 6:** Regional status of cervical lymph node metastasis in 162 cases of PTC.

	Lymph node metastatic level						*χ^2^*	*P* value
	I	II	III	IV	V	VI		
+	3	25	57	56	25	133	302.366	<0.001
–	159	137	105	106	137	29
metastasis rate	1.9%^a^	15.4%^b^	35.2%^c^	34.6%^c^	15.4%^b^	82.1%^d^

For superscript a, b, c and d with the same letters, there was no statistically significant difference between groups (P > 0.05), but there was statistically significant difference between groups with different letters (P < 0.05).

## Discussion

Cervical lymph node metastasis is the most important clinical feature of thyroid cancer recurrence and metastasis. Accurate prediction of lymph node metastasis before treatment plays an important role in selecting reasonable treatment methods, improving curative effects and improving the prognosis of patients. A case-control study (13) based on a large data set showed that local CLNM may increase the unfavorable prognosis of thyroid cancer. Ultrasound has been recommended by several guidelines as the preferred method for evaluating thyroid nodules and cervical lymph nodes; however, conventional preoperative ultrasound is less sensitive to CLNM. Therefore, how to improve the detection ability of CLNM through ultrasound examination before surgery is particularly important. A better preoperative evaluation of the characteristics of the tumor, including size, capsule integrity and multifocality, with ultrasonography in combination with other risk factors, including patient age et al, can provide useful information for the management of patients diagnosed with PTC to determine whether a surgical approach will improve the patient’s survival rate and the postoperative quality of life. In this study, we divided PTC patients into a lymph node metastasis group and a nonmetastasis group and analyzed the clinical characteristics, conventional ultrasound features, and CEUS features of PTC nodules to investigate the potential preoperative predictive performance of CLNM.

The median age of diagnosis for thyroid cancer is approximately 45 years, with women being more likely to be diagnosed with differentiated thyroid cancer than men (14). In this study, the female thyroid cancer incidence was approximately 4.49 times higher than that in men, and there were significant differences in gender and age between the two groups (*P*<0.05). The CLNM rate was higher in men (53.6%) than in women (40.3%). Although women have a higher incidence of thyroid cancer than men, men who are diagnosed with thyroid cancer have a worse prognosis (15). Age ≤45 years was found to be an independent risk factor for lymph node metastasis in patients with PTC (16). This was also confirmed in our research. In addition, age was the most significant predictor of CLNM in our data. Further statistics showed that the probability of lymph node metastasis was 63.4% (26/41) in patients under 30 years old, and 55.2% (80/145) in patients 31 to 45 years old. Although there was no statistically significant difference, it also provided some reference value for clinical practice. We assumed that the younger patients (≤45 years old) with PTC may be more aggressive than older patients (>45 years old) with PTC. In clinical practice, preoperative assessment of lymph node status in young patients should be more careful, and the treatment strategies for younger patients may be different.

Our study found that the size of PTC nodules was associated with CLNM (*P <*0.001), with tumor size ≥1 cm as an independent risk factor for lymph node metastasis, which is consistent with the findings of Kim et al. (11, 12). At the same time, we further divided the nodules into three groups, which were <1 cm, 1-2 cm, >2 cm, the metastasis rates were 29.8% (59/198), 56.3% (81/144) and 59.5% (22/37), respectively (χ2 = 28.517, *P*<0.001), this indicates that the larger the tumor size, the greater the possibility of CLNM. Tumor cells grow through uncontrolled division and proliferation; the larger the tumor is, the faster it grows, and the more likely it is to metastasize (17). Therefore, when the tumor size of PTC is ≥1 cm, and the tumor size is larger, there is a high likelihood of CLNM, and preventive lymph node dissection should be recommended if there are no other risk factors for surgery. Tumor size has become a reference index for tumor staging and assessing surgical ranges (18). Therefore, for suspected malignant thyroid nodules, it is helpful to have an accurate measurement of tumor size in preoperative ultrasonography.

Our study showed that microcalcifications in thyroid nodules were significantly different between the metastasis and nonmetastasis groups (*P*<0.05), which is consistent with the findings of previous studies (19). Microcalcification occurs when calcium salt deposits form due to excessive tumor tissue proliferation, and it is commonly used as a potential malignant feature on ultrasonography (20). In addition, some scholars have pointed out that the sand precursor in the tumor cytoplasm is the main reason for the formation of microcalcification foci in thyroid tissue, confirming that the sand precursor represents the active biological process of tumor cells, which are shown as microcalcification foci during ultrasound examination (21). Microcalcification is more common in PTC than in other types of thyroid cancer. However, pathological microscopic collagen, fibrosis and a small number of glia can be confused with microcalcifications by strong echo of microspots on ultrasound. Therefore, the correlation between them and lymph node metastasis needs further study. As previously reported (22), microcalcification was an independent predictor in PTCs with CLNM. Although we did not obtain such results, microcalcification was significantly correlated with CLNM in univariate analysis. The main reasons might have been to the sample size.

Kamaya et al. proposed four US characteristics of thyroid cancer invading the capsule, including nodule contact with the capsule, outward protrusion of the capsule outline, interruption of the capsule integrity, and microcirculation of the nodule extending out of the capsule (23). CEUS has a higher sensitivity and specificity for capsule invasion than conventional ultrasound. If a loss of capsule continuity is shown on CEUS, it can be used to predict CLNM in patients with PTC (24). In the present study, we found statistically significant differences in capsule integrity and thyroid nodule contact with capsule between the lymph node metastatic and non-metastatic groups(*P*<0.05). WANG (25) et al. indicated that capsule invasion more than 1/4 perimeter of PTC became an independent risk factor of central cervical neck lymph node metastasis. In contrast, our research assessed cervical lymph node metastasis and was not limited to central lymph nodes, and we further quantified the invasion of the capsule and divided it into four groups according to the length of transverse or longitudinal invasion of the capsule. The results indicated that the nodules had more contact with the capsule, CLNM was more likely to occur. A contact range of the adjacent capsule >50% is an independent risk factor for CLNM. It is likely that when the capsule contact is greater, the chance of the capsule being invaded by the tumor is higher. When the tumor invades the surrounding tissue *via* the interrupted capsule, CLNM occurs.

Gland metastases often occur at an early stage in patients with PTC, and multifocality is an important independent risk factor for CLNM (26). Our study indicated that multifocality is also a risk factor for CLNM in PTC (*P<*0.05), which is consistent with the findings of previous studies (27). The OR value of multifocality (OR=2.370) in our study was higher than that (OR=1.297) reported by Liu et al. (28). Whether the occurrence of multifocality is due to intraglandular metastasis or is of polyclonal origin is still controversial. The former theory suggests that multifocal cancers originate from a single focal disease that spreads through the abundant lymph system in the gland; therefore, other smaller satellite foci are formed *via* lymph system spreading. The latter theory suggests that multifocal cancers are relatively independent and not related to each other. In our research, CLNM was more likely to occur in patients with multifocal PTC, which may be due to the rich lymphatic network in the thyroid gland, and multifocality may be due to tumor cell metastasis in the gland *via* lymphatic vessels. Therefore, CLNM is more likely to occur.

Previous studies found that the enhancement patterns of PTC on CEUS are a useful tool to predict CLNM (29, 30). Hong (31) et al. showed that hyperenhancement or iso-enhancement could be used as an independent risk factor to predict the existence of CLNM. In our present study, univariate analysis showed that the CLNM rate in the hyper-enhancement group (67.7%, 21/31) was higher than that in the other two groups (40.7%, 40.0%), and the difference was significant. This difference is likely due to the difference in the blood vessel network in different patients. Hyperenhancement often indicates that tumors are rich in nourishing blood vessels, and angiogenesis is related to tumor growth and proliferation, making tumors more aggressive and prone to lymph node metastasis (32). However, in our multivariate analysis results, hyperenhancement was not an independent predictor for predicting CLNM, and may due to the inconsistency between different observers and the difference in parameters of the ultrasonic machine. The enhancement intensity may be different when judging the qualitative indicators and other results.

Gong (33) et al. reported that most CLNMs in patients with PTC are at level VI, and lateral CLNMs are mainly located at levels II-IV. Our study showed that CLNM mainly occurred at level VI, and most of the lateral cervical metastases were at levels III and IV, with frequencies of 82.1%, 35.2%, and 34.6%, respectively. Level VI is the anterior cervical lymph node group, also known as the central lymph node, which is the sentinel lymph node of thyroid cancer lymph node metastasis. In the Chinese guidelines (34) for the diagnosis and treatment of thyroid nodules, central lymph node dissection should be performed routinely for thyroid nodules diagnosed as PTC. Therefore, the preoperative diagnosis of lateral cervical lymph nodes is more important. Preoperative localization and qualitative and quantitative diagnosis of cervical metastatic lymph nodes in levels II, III, IV, V and VI are helpful for surgeons to develop a better preoperative lymph node dissection plan, reduce the scope of unnecessary dissection, shorten operation time and achieve the best efficacy.

In this study, we found the combination of four features (tumor size, age, multifocality and contact range of the adjacent capsule >50%) showed great performance in predicting CLNM. Using ROC curve, we identified the AUC was 0.756. And this nomogram lymph node metastasis prediction model proposed in our research, combining independent risk factors, provides a visual tool for surgeons and sonographers to predict lymph node metastasis in PTC patients before surgery. Previous studies (35) have shown that nomograms have been helpful in dealing with dilemmas in colorectal cancer, but they have rarely been reported for predicting CLNM risk in PTC patients. The results of our nomogram model performed well, and we believe that it can be used as a valuable diagnostic tool for CLNM in PTC patients.

There are some limitations to this study. First, we used retrospective data from a single institution, which may introduce some bias and affect the applicability and generality of the results. In the future, we will conduct prospective studies to solve this problem. Secondly, the relevant factors we explored are still not comprehensive, and future studies will need to include more thyroid nodule location information and clinical laboratory indicators. Finally, the nomogram needs further validation in a large polycentric cohort of PTC patients. Despite these shortcomings, the risk predictors that made up the nomogram of this study could be obtained preoperatively with good identification ability and satisfactory performance of internal validation.

In conclusion, our study indicated that the characteristics of PTC on ultrasonography and CEUS can be used to predict CLNM to some degree. We developed a diagnostic model that includes all adverse factors. This method can be used to help screen patients with a high risk of CLNM and assist clinicians in deciding whether surgical dissection of cervical lymph nodes is indicated in consideration of improving patients’ postoperative quality of life.

## Data Availability Statement

The raw data supporting the conclusions of this article will be made available by the authors, without undue reservation.

## Ethics Statement

The studies involving human participants were reviewed and approved by Ethics Committee of the First Hospital of Shanxi Medical College (2018LL139). The patients/participants provided their written informed consent to participate in this study. Written informed consent was obtained from the individual(s) for the publication of any potentially identifiable images or data included in this article.

## Author Contributions

L-PL designed the study. J-JL, Y-HH, Y-PS, X-XZ, Y-JZ and Y-FZ collected the clinical data, ultrasonography images and histopathological/cytopathology results. Y-PS and X-XZ processed the clinical and images data. TX, CL and Y-PS performed the statistical analysis. TX and CL drafted the manuscript. L-PL, TX, CL, and J-JL revised the manuscript. All authors contributed to the article and approved the submitted version.

## Funding

This work was supported by Application Basic Research Project of Science and Technology Department of Shanxi Province (201801D121340), and Key Research and Development Program of Science and Technology Department of Shanxi Province (201903D321190).

## Conflict of Interest

The authors declare that the research was conducted in the absence of any commercial or financial relationships that could be construed as a potential conflict of interest.

## Publisher’s Note

All claims expressed in this article are solely those of the authors and do not necessarily represent those of their affiliated organizations, or those of the publisher, the editors and the reviewers. Any product that may be evaluated in this article, or claim that may be made by its manufacturer, is not guaranteed or endorsed by the publisher.
